# Deep Learning for Combating Misinformation in Multicategorical Text Contents

**DOI:** 10.3390/s23249666

**Published:** 2023-12-07

**Authors:** Rafał Kozik, Wojciech Mazurczyk, Krzysztof Cabaj, Aleksandra Pawlicka, Marek Pawlicki, Michał Choraś

**Affiliations:** 1 Faculty of Telecommunications, Computer Science and Electrical Engineering, Bydgoszcz University of Science and Technology, 85-796 Bydgoszcz, Poland; 2 Institute of Computer Science, Division of Software Engineering and Computer Architecture, Warsaw University of Technology, 00-661 Warsaw, Poland; 3 Faculty of Applied Linguistics, University of Warsaw, 00-927 Warsaw, Poland

**Keywords:** deep learning, fake news, ensemble of classifiers, text classification, misinformation

## Abstract

Currently, one can observe the evolution of social media networks. In particular, humans are faced with the fact that, often, the opinion of an expert is as important and significant as the opinion of a non-expert. It is possible to observe changes and processes in traditional media that reduce the role of a conventional ‘editorial office’, placing gradual emphasis on the remote work of journalists and forcing increasingly frequent use of online sources rather than actual reporting work. As a result, social media has become an element of state security, as disinformation and fake news produced by malicious actors can manipulate readers, creating unnecessary debate on topics organically irrelevant to society. This causes a cascading effect, fear of citizens, and eventually threats to the state’s security. Advanced data sensors and deep machine learning methods have great potential to enable the creation of effective tools for combating the fake news problem. However, these solutions often need better model generalization in the real world due to data deficits. In this paper, we propose an innovative solution involving a committee of classifiers in order to tackle the fake news detection challenge. In that regard, we introduce a diverse set of base models, each independently trained on sub-corpora with unique characteristics. In particular, we use multi-label text category classification, which helps formulate an ensemble. The experiments were conducted on six different benchmark datasets. The results are promising and open the field for further research.

## 1. Introduction

Today, the level of expertise, education, or experience is no longer an obstacle to spreading opinions and becoming a content creator. Through the Internet, content creators are able to remain fully anonymous if they so choose. Nevertheless, this situation has laid the groundwork for the fake news trend to surface. Though the disinformation problem is as old as modern civilization, the evolution of digital media has dramatically transformed the way deception is spread. Eventually, it became a highly influential weapon.

With the evolution of machine learning and, in particular, the development of deep learning NLP-based methods, an array of new tools has been proposed to combat fake news problems [1,2,3].

However, most of the analyzed solutions propose a monolithic approach that focuses on fake news detection as a single-task learning problem in which the entire ML model is trained mainly from scratch [4,5,6]. Moreover, only some approaches consider domain segmentation before fake news detection, as, typically, the existing methods mix different types of models for feature extraction and other classification solutions.

Therefore, in this paper, the authors put forward a more scalable solution where a committee of classifiers is composed to address the problem of fake news detection. In more detail, the authors propose an alternative, novel approach in which the construction of a diversified pool of base models is learned independently on a sub-corpus of texts with unique characteristics (resulting directly from multi-label classification). This improves the detection efficiency of fake news in textual form.

The paper is structured as follows. First, the related work is discussed in Section 2. Next, in Section 3, the authors introduce the framework for the proposed deep learning solution. The experimental evaluation results are included in Section 4. Finally, the paper closes with final remarks, at the same time outlining the perspectives on future work in Section 5.

## 2. Related Work

At the beginning of this millennium, it has started to become evident that the diversification of sources providing online news, as well as the utilization of social network sites to filter and consume them, may lead to the risk of containing its users within inevitable “bubbles” where only information that goes along with their intuition is presented to them [7]. Moreover, online opinion leaders have begun to appear and increasingly influence online communities, often using fake news [8].

The rapid expansion of fake news has put more pressure on legitimate news sources to ensure reliable information and create tools to verify the presented facts. In this vein, various projects started to appear. Firstly, fact-checkers, i.e., specialized websites, have been widely adopted by consumers. This includes, for instance, FactCheck.org in the USA, Maldita.es in Spain, Demagog.org.pl in Poland, and FactCheck Initiative in Japan. These websites allow for, e.g., the presentation of multimedia content metadata so anyone can verify its originality. However, there are better solutions than this, as metadata can be easily modified, and there is no information on what has been changed in such content. Secondly, there are solutions like Trustproject (https://thetrustproject.org/), where over 120 news organizations, including well-known media companies such as the BBC, South China Morning Post, and Bay Area News Group, are working towards greater transparency and accountability in the global news industry. Trustproject provides a protocol encompassing eight indicators of trust. For example, one of them concerns the capability of the news addressee to assess the journalist’s level of expertise. Although news consumers have welcomed solutions like Trustproject, it must be noted that their procedures still need to be automated owing to the lack of suitable tools.

Apart from the above, many technological developments have also been used to try to detect fake news. These solutions have already been analyzed in several surveys on this topic, e.g., in [9,10,11].

One such solution is digital watermarking, which may be used to overcome issues such as copyright protection, content authentication, detection of tampering, and so on [12]. For example, one of the watermarking applications is fingerprinting, which allows one to apply a unique fingerprint, which may then be utilized to identify the recipient of the content; this is true for each individual copy of the disseminated content. Owing to that, it becomes possible to deter illegal redistribution as it makes it possible for the content owner to find out where the redistributed copy came from [13,14]. However, it has been proved that the application of digital watermarking techniques is of great value in the domain of fake news identification and tracing; this line of research is currently pursued within the DISSIMILAR project [15].

Many works have been devoted to tackling the problem of fake news detection in various types of content (text, multimedia, etc.) using AI-based solutions.

At present, the research community is casting a discerning eye on one particular form of fake news: deepfakes. In these fake videos, it is practically impossible to distinguish whether they are forged or not. This brings an urgent need to create automated detection methods. The existing literature on this subject discusses several attempts at countering deepfakes [16] or fake news in images [17]. Conventionally established methodologies for identifying counterfeit videos center on discerning the subtle features present within these manipulated recordings. For instance, the technique introduced in [18] relies on identifying eye blinking, as this physiological signal is typically incorrectly mapped in synthesized fake videos. Conversely, the approach presented in [19] offers a visualization of the CNN layers and filters, demonstrating that the eyes and mouth are instrumental in identifying faces tampered with by prevailing deepfake software utilities [20,21]. A recent paper presents another solution, i.e., DeepTag, an end-to-end deep watermarking framework incorporating a GAN simulator that can employ common distortions to facial images [22]. Consequently, one can extract the watermark to discern the original, untouched facial image.

It is also worth noting that to accelerate advancements in the detection of fake media, the DFDC (DeepFake Detection Challenge) dataset [23] was created and released by several major industry players like Amazon Web Services (AWS), Facebook, Microsoft, and the Partnership on AI’s Media Integrity Steering Committee and academics. The main aim of this challenge is to boost research to design and develop innovative new solutions to expedite deepfakes and manipulated media detection.

Additionally, significant research has been devoted to detecting fake news in textual information. In [24], for instance, the authors introduce an innovative model for automated fake news credibility assessment. Leveraging a combination of overt and latent attributes derived from text, they establish a deep diffusive network model that concurrently learns representations for news pieces, their authors, and topics.

In [4], Khan et al. investigate the performance of various classical machine learning techniques and neural network models for textual fake news detection using three different datasets. They concluded that the best-performing solution was Naive Bayes with n-gram (bigram TF-IDF) features.

Next, in [5], the authors simultaneously analyze the correlations of publisher bias, news stance, and relevant user engagements. Then, based on these observations, they introduce a novel fake news detection framework. The experimental results obtained on two comprehensive real-world fake news datasets prove the effectiveness of the proposed approach.

Another fake news detection approach is described in [6]. The authors use propagation features to detect fake news on Twitter. They note that real news is significantly larger than fake news and is typically spread by users who have been active on Twitter for a long time and have more followers and fewer followings. Then, using these features, they train a Random Forest classifier, which results in 87% accuracy. Finally, they also use Geometric Deep Learning solutions and create a graph neural network that directly learns from the propagation graphs and results in 73.3% accuracy.

Huh et al. [25] unveil a learning algorithm adept at identifying visual alterations in images. Remarkably, it is trained solely on a substantial collection of authentic photographs and capitalizes on the inherent photo EXIF metadata as the guiding beacon for model training. This allows for evaluating if an image is produced by a single imaging pipeline. The experimental analysis revealed that the proposed approach achieves state-of-the-art performance on the chosen image forensics benchmark datasets.

In [1], the authors present a deep hierarchical co-attention network. This network is designed to learn feature representations for fake news detection and to identify relevant sentences or comments. The experimental evaluation conducted on real-world datasets showed that the proposed framework is effective. Another attention-based approach was proposed in [26].

Then, in [27], another approach to supervised learning-based fake news detection is presented. Apart from analysing the usability of features proposed in the literature, the authors point to a new set of features and evaluate the prediction performance of existing solutions and features for performing automatic detection. The obtained experimental results prove that the prediction performance of the utilized features in combination with state-of-the-art classifiers has suitable discriminative power for detecting fake news.

In [28], Perez-Rosas et al. discuss the automatic identification of textual fake news. Apart from proposing a new detection approach capable of achieving accuracy ca. 76%, the authors also introduce two datasets that can help the research community in benchmarking fake news detection methods. Additionally, the paper also includes the results of a comparative analysis of the automatic and manual identification of fake news.

A unique strategy for fake news detection was introduced in [28]. This method merges the publishing and sharing patterns of both publishers and users to enhance the accuracy of detecting fake news and predicting its credibility. When tested on three actual datasets, the method showcased its capability by achieving a detection accuracy surpassing 91%.

The following solution to fake news detection proposed in [29] emphasizes considering relational features like sentiment, named entities, and facts extracted from structured and unstructured data. Based on the presented experimental evaluation, the obtained results are generally better for all tested classifiers.

O’Brien et al. [2] try to overcome a lack of transparency in the decision-making process (black-box problem) of deep neural networks by showing that the emergent representations of such networks can pinpoint subtle differences between fake and real news. This makes convolutional neural networks a powerful tool for detecting fake news. The authors’ evaluation also shows the generalization capabilities of such solutions in detecting fake news in novel subjects based solely on language patterns.

The UMLARD model was proposed in [30]. It focuses on rumor detection. In particular, the authors tackled the problem of learning representations from different user perspectives. They utilized a fusion mechanism to enhance prediction. The authors reported performance improvements with respect to various baseline methods.

In [31], the researchers introduce a Deep Normalized Attention-based mechanism designed to enhance the extraction of dual emotion features. The proposal also uses Adaptive Genetic Weight Update-Random Forest (AGWu-RF), which is utilized for the classification. Similarly, in [32] the authors propose a novel rumor detection model called graph contrastive learning with feature augmentation (FAGCL). This technique allows the author to inject noise into the feature space, which enables the model to learn contrastively by constructing asymmetric structures.

A fast fake news detection model was proposed in [33]. This approach is particularly focused on cyber–physical social services. The authors mostly rely on Chinese texts. Moreover, they argue that fake news items are generally short texts and can be effectively accompanied by relevant keywords. In order to extract feature vectors for the analyzed texts, a convolution neural network (CNN) is facilitated.

In the quest to combat misinformation, traditional methods often rely on intricate features or credibility networks, requiring expert knowledge and extensive engineering. Recent advances in deep learning have offered promising approaches, but they struggle with over-reliance on content features and neglect the individual user’s impact in spreading rumors. Addressing these challenges, the proposed UMLARD model (User-aspect Multi-view Learning with Attention for Rumor Detection) effectively captures different user perspectives, combines them using a distinctive fusion mechanism, and integrates these learned features with content features for improved rumor detection. Experiments on real-world datasets demonstrate that UMLARD surpasses existing methods, providing both enhanced performance and interpretability.

Finally, in [34], the authors develop a deep learning-based system for concept extraction and relation identification. The solution is based on the BERT ensemble using a majority voting strategy.

The analysis of the state-of-the-art reveals various limitations of the existing methods, which the authors intend to address in this work. These can be summarized as follows:

- Observation: The majority of the analyzed solutions propose a monolithic approach that focuses on fake news detection as a single-task learning problem (STL), where the entire ML model is trained mainly from scratch.
  Proposed solution: In this paper, the authors propose a more scalable solution where a committee of classifiers is composed to address the fake news detection problem.
- Observation: Very few approaches consider domain segmentation prior to fake news detection. Instead, the authors frequently mix different types of models for feature extraction (e.g., RNN, CNN) and different classification methods (e.g., DNN, SVM, RF, etc.).
  Proposed solution: In this approach, a diversified pool of base models is considered that were trained independently on a sub-corpus of texts with unique characteristics.

## 3. Proposed Solution

In this section, the authors outline the architecture of the approach proposed in this paper. Then, they characterize how data harvesting and open media crawling are typically performed. Finally, the utilized document representation and classification are described.

### 3.1. Architecture

**Figure 1:**
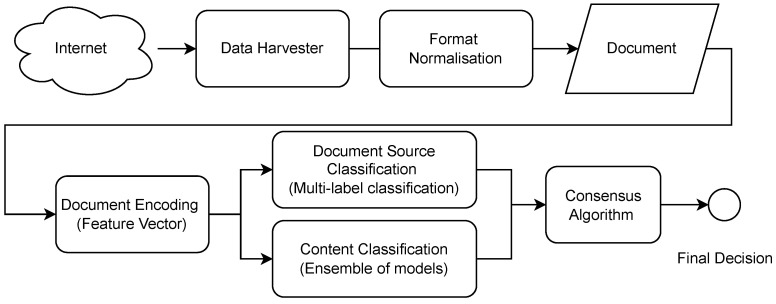
Information flow used by the proposed approach.

The architecture of the proposed solution is presented in Figure 1. The textual data were collected from open data sources (e.g., news providers) utilizing data harvesters (described in Section 3.2). Next, the collected data were normalized regarding the format so that all documents were stored in a similar and coherent way (e.g., title, document, body, author, and date when the article was published). It must be emphasized here that the feature vectors (the document encoding step) are extracted only from the document body. This way, one can avoid judging the document by its author or origins. The extracted features rely on the BERT language model described in Section 3.4. The novelty of the architecture is the fact that the document source is predicted, which drives the votes obtained by several (domain-related) content classification models.

### 3.2. Data Harvesting and Open Media Crawling

In fake news-related research, an important question is always associated with data sources. One solution that could be utilized for this purpose is media crawling, often called harvesting. In this technique, specialized software, which behaves as a browser with custom logic, automatically visits a predefined list of resources on the Internet and downloads certain content (e.g., files) for further analysis. These solutions have been used during various kinds of research for years. The first papers describing such systems in the IEEExplore database are from the late 1990s, for example, [35,36].

Access to data gathered by web crawlers can be achieved by various means. A short survey presented in [37] defines three methods of how a researcher can obtain data via web harvesters. The first one utilizes dedicated extensions to the web browser, which record some valuable information to JSON, XLS, CSV, or HTML files. Currently, such a solution can be used within Firefox and Google Chrome browsers. The second solution is related to dedicated Open Source, commercial, or is even delivered as SaaS products. The provided software harvests all previously defined data sources. The third method utilizes some auxiliary tools and libraries that the researcher can use to develop a custom harvester specialized in the nature of the conducted research. This solution requires the highest effort from the three ways mentioned above. However, it also gives the best results. Further on in this section, the latter kind of solution is discussed.

**Figure 2:**
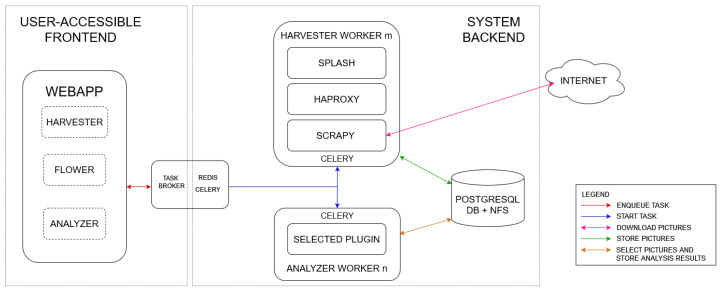
Architecture of the harvester, which consists of front-end (for administration) and back-end (for data harvesting) parts. The core of the system utilizes Celery task distribution framework, which controls data crawling process.

Figure 2 presents the architecture of a custom harvester which was developed during the DISSIMILAR project. The system consists of two standard parts: (i) the front-end and (ii) the back-end.

While the front-end is designed for system administration, the back-end focuses on data crawling. The connection to web servers is performed via Scrapy library (https://scrapy.org/, accessed on 21 October 2022). Since modern web pages, in most cases, are dynamically created using JavaScript, Splash framework (https://splash.readthedocs.io/en/stable/, accessed on 21 October 2022) is used to execute scripts and provide the final version of the generated HTML code.

In order to have control over the harvesting process, we developed the processing pipeline on top of the Celery task distribution subsystem. This allows for controlling dedicated workers delegated to harvesting and analyzing processes. Moreover, such an architecture enables the distribution of web scraping on multiple machines.

### 3.3. BERT-Based Document Representation and Classification

As part of the research, a collection of benchmark sets (commonly used by various researchers) was compiled. This allowed us to create an integrated language corpus that eventually mixed various categories of content (e.g., politics, health, news, etc.). Because the datasets differ in terms of various characteristics (e.g., text length), there is a separate problem related to the extraction of features from documents.

**Figure 3:**
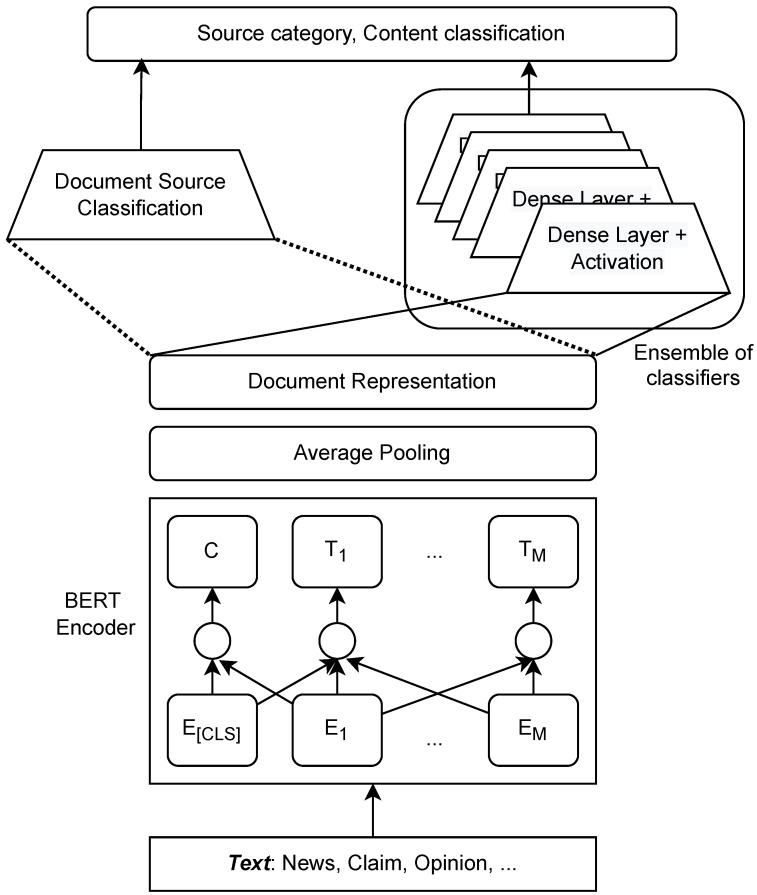
Document representation and classification model.

To address this, a unified and common methodology for all datasets’ feature extraction was adopted. In particular, the same BERT language model (see Figure 3) was adapted to establish word, sentence, and document representation. In that regard, each input sentence is first transformed by a tokenizer, which breaks the sequence of words into individual tokens and then adds unique [CLS] (Classification) and [SEP] (Separator) tokens at the beginning and the end, respectively. Moreover, each token is replaced with the corresponding unique identifiers, which are established from the so-called embedding table. To simplify the text processing and classification pipeline, the tokenizer is configured to either truncate or pad sentences to a maximum of 512 tokens.

In this paper, the authors adapted the pre-trained DistilBERT model, which is a ’lightweight’ variant of BERT. While training the entire model (DistilBERT + classification head), the BERT layers are left frozen during this process, and only the weights in the classification head are updated. In order to decrease the feature vector dimension, average pooling over the tokens returned by the BERT model was applied.

### 3.4. Document Source Classification

As is visible in Figure 3, on top of the document representation layer are two classification pathways. The right pathway is actually a committee of classifiers. In this case, each model in the ensemble is specialized in a different type of document. The one on the left is responsible for document source classification. In this paper, we used six different datasets, as detailed in Section 4.2, and therefore, each dataset is considered as a different source. The role of the document source classifier is to build consensus in the community in the above-mentioned processing pathway. More precisely, the classifier outputs the weights which are assigned to the responses of the base models. For example, if we have a document entirely devoted to COVID, (most likely) the document source classifier will delegate the prediction (will associate the highest weight) to the responses provided by the base model, which has competencies in this domain. Moreover, we used a diverse set of base models that we independently trained on sub-corpora with unique characteristics. Therefore, (from our perspective) multi-label text category classification is the entity that formulates the abovementioned ensemble.

## 4. Results

First, this section describes the data and methodology used to conduct the experimental evaluation. Next, the results for a single dataset are presented. Then, the outcomes of the text source classification accuracy are outlined. Finally, the authors focus on the results from the ensemble of BERT models.

### 4.1. Methodology and Tools Used for Experiments

In order to compare different approaches and configurations on different datasets, the 5x2-fold cross-validation method was employed. In this method, conventional two-fold cross-validation is utilized, with outcomes being averaged. Additionally, the standard deviation from the mean is computed to highlight both the variability and the importance of the distinctions. The 5x2-fold cross-validation (CV) method’s nested structure helps achieve a more robust model. The outer loop helps assess how well the model generalizes across different subsets of the data, and the inner loop (two-fold CV) can help reduce the variance in estimates compared to a single-fold validation.

To assess the performance, we used common metrics for comparing classification effectiveness, namely the following:

- Precision = $\frac{TP}{TP+FP}$.
- Recall (Sensitivity) = $\frac{TP}{TP+FN}$.
- Specificity = $\frac{TN}{TN+FP}$.
- Balanced Accuracy = $\frac{Sensitivity+Specificity}{2}$.
- F1-score = $2*\frac{Precision*Recall}{Precision+Recall}$.
- G-mean = $\sqrt{Sensitivity*Specificity}$.

In the above equations, TP, FP, FN, and TN indicate True Positive, False Positive, False Negative, and True Negative, respectively. TP (True Positive) is the number of fake news cases correctly classified as such. FN (False Negative) is the number of fake news cases incorrectly classified as real news. FP (False Positive) is the number of real news cases that are incorrectly identified as Fake, and TN (True Negative) is the number of real news cases correctly classified as such.

### 4.2. Data Used for Experiments

Six different datasets related to the fake news detection problem were used in all experiments. The details of the datasets, as well as the numbers they will be referred to as in this paper, are presented in Table 1.

### 4.3. Single Dataset Performance

In this subsection, for the classification, we report the accuracy obtained with a BERT-based classifier (as defined in Section 3) on the considered datasets. The results are reported in Table 2. In all cases, the authors repeated five times two-fold cross-validation (5x2 CV) on a single dataset. The presented outcomes are shown as averages along with the standard deviation from the mean value. In the case of the *F1*, *Precision*, and *Recall* metrics, a weighted average calculated on both classes (fake news and real news) is reported. Here, it can be noticed that for all datasets, the BERT model achieves relatively high values of performance metrics (e.g., Balanced Accuracy exceeds 75%).

**Figure 4:**
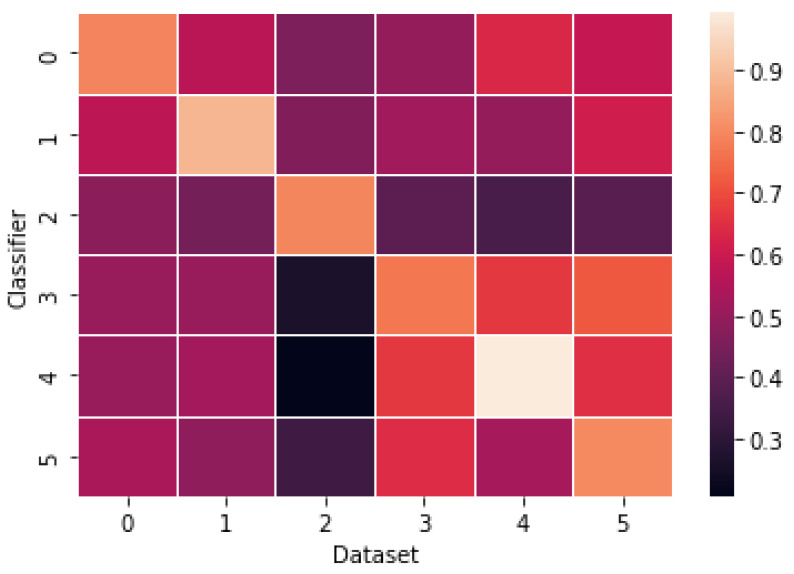
Balanced Accuracy achieved with BERT classifier (pre-trained on a single dataset and evaluated on another: 0—CovidFN, 1—MMCovid, 2—QProp, 3—ISOT, 4—GRAFN, 5—PubHealth).

In such cases, the results can be promising and considered optimistic. However, the truth is that the model needs to be better generalized to other datasets. It is quite vivid when observing Figure 4. The diagram depicts the base model’s performance pre-trained on one dataset and evaluated on the other. As a pre-trained model, one understands the best model amongst all validation folds. The bright diagonal in Figure 4 indicates that the classifier performs well on the original dataset. One can also notice some brighter areas, which may suggest that some datasets share similar contents (e.g., ISOT and GRAFN, which are highly populated with political news).

### 4.4. Text Source Classification Accuracy

In Table 3, various metrics for multi-label source classification are presented. As in the previous cases, the 5x2 cross-validation was employed to compare and report the classification performance. The goal of the multi-label classification model is to correctly recognize the dataset (the source of the analyzed text). The worst results were obtained for QProp, while for the other datasets, performance metrics achieved relatively high values (e.g., F1 scores close to 90%). In Table 3, the Accuracy, Balanced Accuracy, and the G-mean are reported only as an average because these involve all labels when calculating their values (e.g., Balanced Accuracy requires an average of recall scores per class to be calculated).

### 4.5. Ensemble of BERT Models

Finally, in this subsection, the results obtained for the ensemble of BERT models are showcased. More precisely, the ensemble is constructed of a diversified pool of base models which learned independently from a sub-corpus of texts. The unique characteristics result from multi-label classification, which was presented in the previous section.

In Table 4, the authors compare the proposed approach to other techniques known from the literature, namely the following:

- Batch MTL—a single classification model is trained from scratch on the entire dataset.
- Majority Voting—an ensemble of the BERT model is constructed, and the consensus is established using a hard voting approach (e.g., [34]).
- Weighted Voting—an ensemble of BERT models combined with a soft voting approach (e.g., [44]).

Based on the results presented in Table 4, it is visible that the method proposed in this paper outperformed other state-of-the-art solutions. This demonstrates the potential of the proposed approach. Concerning the Balanced Accuracy, the introduced technique turned out to be better by almost 30% than the Weighted BERT Ensemble and 5% better than Batch MTL. Regarding the average F1 metric, the improvement over the Weighted BERT Ensemble is even more remarkable, i.e., about 50%, but at the same time comparable to Batch MTL (0.8% increase).

## 5. Conclusions

Advanced deep learning techniques offer potent tools for addressing the issue of false news. However, these systems often struggle with poor model generalization in real-world scenarios due to data limitations. Unfortunately, many existing solutions adopt a monolithic approach, treating fake news detection as a single-task learning problem and utilizing a variety of models and classification methods without considering domain segmentation.

To overcome these limitations, our paper proposes an innovative solution involving a committee of classifiers in order to tackle the fake news detection challenge. In that regard, we introduce a diverse set of base models, each independently trained on sub-corpora with unique characteristics. In order to evaluate different approaches and configurations on the considered datasets, we employed the 5x2-fold cross-validation (CV) method. This nested CV structure enhances model robustness by assessing generalization across different data subsets and reducing variance in estimates.

The experimental results (on six different benchmark datasets) show that the suggested approach is promising, opening further research areas. In particular, the proposed approach outperforms the Weighted BERT Ensemble by nearly 30% in Balanced Accuracy and exhibits a 50% improvement in the F1 metric. It also sustains competitiveness with Batch MTL, with a slight 0.8% increase in the F1 score.

The promising results and performance indicate the future direction and value of using ensembles of classifiers in misinformation detection systems.

## Figures and Tables

**Table 1:** Summary of datasets related to news authenticity.

No.	Dataset Name	Size	Content Type	Description	Reference
1	COVID-19 Fake News	10,200 items (∼9700 fake, ∼500 real)	News (COVID-19)	Fake and legitimate news items related to COVID-19.	[38]
2	MM-Covid	11,000 items (∼4000 fake, ∼7000 real)	News (Multilingual)	Fake and legitimate news items with social context.	[39]
3	Q-Prop	50,000 articles	News	Articles labeled as “legitimate” or “propaganda”.	[40]
4	ISOT	44,898 documents (23,481 fake, 21,417 real)	News	Trustworthy and fake documents with additional metadata.	[41]
5	GRAFN	13,000 posts	News (Global Politics)	Text and metadata from 244 websites.	[42]
6	PubHealth	Not specified	Health (Public /Biomedical)	Documents with labels and explanations related to public health topics.	[43]

**Table 2:** Performance of BERT-based classifier trained and evaluated on the same datasets (results reported using 5x2-fold cross-validation methodology).

Dataset	Bal. Acc.	F1	G-Mean	Precision	Recall
Covid-FN	87.6 ± 0.8	91.9 ± 0.4	87.0 ± 0.9	92.2 ± 0.3	92.2 ± 0.3
MMCovid	78.8 ± 0.5	80.2 ± 0.5	78.6 ± 0.5	80.2 ± 0.5	80.2 ± 0.5
QProp	77.3 ± 1.3	96.7 ± 0.2	74.1 ± 1.8	96.7 ± 0.2	97.0 ± 0.2
ISOT	76.7 ± 0.4	92.6 ± 0.1	73.8 ± 0.6	92.5 ± 0.1	93.1 ± 0.1
GRAFN	99.3 ± 0.0	99.2 ± 0.0	99.3 ± 0.0	99.2 ± 0.0	99.2 ± 0.0
PubHealth	79.9 ± 0.2	88.5 ± 0.1	78.4 ± 0.2	88.4 ± 0.1	88.9 ± 0.1

**Table 3:** Performance of the proposed text source classification model (dataset name is considered here as a label returned by the model).

	F1	Precision	Recall
CovidFN	97.5 ± 0.1	97.2 ± 0.2	97.8 ± 0.2
MMCovid	88.7 ± 0.3	89.6 ± 0.7	87.8 ± 0.3
QProp	14.2 ± 0.9	45.6 ± 3.4	8.4 ± 0.6
ISOT	92.7 ± 0.1	91.8 ± 0.2	93.7 ± 0.1
GRAFN	92.1 ± 0.1	91.4 ± 0.1	92.8 ± 0.2
PubHealth	87.0 ± 0.2	87.9 ± 0.4	86.1 ± 0.4
Average	91.0 ± 0.1	90.9 ± 0.1	91.5 ± 0.1
Accuracy	91.5 ± 0.1		
Balanced Accuracy	77.8 ± 0.1		

**Table 4:** Classification performance of BERT model ensembles integrated using text source classifier.

Approach	Balanced Accuracy	Avg. F1
Proposed Ensemble Method	82.7 ± 0.1	84.8 ± 0.1
Batch MTL	77.7 ± 0.1	84.0 ± 0.1
Majority Voting BERT Ensemble	65.7 ± 0.1	67.3 ± 0.1
Weighted BERT Ensemble	54.4 ± 0.1	33.5 ± 0.2

## Data Availability

No new data were created or analyzed in this study.
